# CD4+ T Cell Effects on CD8+ T Cell Location Defined Using Bioluminescence

**DOI:** 10.1371/journal.pone.0016222

**Published:** 2011-01-20

**Authors:** Mitra Azadniv, William J. Bowers, David J. Topham, Ian N. Crispe

**Affiliations:** 1 David H. Smith Center for Microbiology and Immunology, Aab Institute for Biomedical Research, University of Rochester Medical Center, Rochester, New York, United States of America; 2 Department of Neurology, Center for Aging and Developmental Biology, Aab Institute for Biomedical Research, University of Rochester Medical Center, Rochester, New York, United States of America; 3 Seattle Biomedical Research Institute, Seattle, Washington, United States of America; New York University, United States of America

## Abstract

T lymphocytes of the CD8+ class are critical in delivering cytotoxic function and in controlling viral and intracellular infections. These cells are “helped” by T lymphocytes of the CD4+ class, which facilitate their activation, clonal expansion, full differentiation and the persistence of memory. In this study we investigated the impact of CD4+ T cells on the location of CD8+ T cells, using antibody-mediated CD4+ T cell depletion and imaging the antigen-driven redistribution of bioluminescent CD8+ T cells in living mice. We documented that CD4+ T cells influence the biodistribution of CD8+ T cells, favoring their localization to abdominal lymph nodes. Flow cytometric analysis revealed that this was associated with an increase in the expression of specific integrins. The presence of CD4+ T cells at the time of initial CD8+ T cell activation also influences their biodistribution in the memory phase. Based on these results, we propose the model that one of the functions of CD4+ T cell “help” is to program the homing potential of CD8+ T cells.

## Introduction

Immune responses against viruses and intracellular pathogens are often delivered by CD8+ cytotoxic effector cells, which kill the pathogen-infected cell and generally the pathogen too. However CD4+ “helper” T cells contribute to the function of CD8+ T cells in such infections. A striking example is found in Hepatitis C Virus infection, where the capacity of both humans and chimpanzees infected with the virus to achieve suppression of viremia is strongly correlated with the strength and diversity of the CD4+ T cell response [1], [2], [3].

The mechanism of CD4+ T cell help in CD8+ T cell responses is complex, and incompletely understood. One important mechanism of action may be *via* an interaction with antigen-presenting cells, termed licensing. In this interaction, the CD4+ T cell undergoes antigen-specific interaction in which it delivers CD40-mediated signals that promote the maturation and function of an antigen-presenting cell [4]. This cell can subsequently induce full activation in a CD8+ T cell that recognizes an epitope of the same antigen, or a distinct antigen expressed on the same particle or cell fragment which the antigen-presenting cell has endocytosed (termed: linked recognition). The licensing interaction can be conveyed *via* cell surface molecules such as CD40, or by soluble cytokines such as IL-2 and IL-12 [5]. An alternative mechanism is the delivery of signals direct from the CD4+ T cell to the CD8+ T cell, if they have been approximated by interaction with the same antigen-presenting cell (termed: the three cell cluster) [6].

After full activation, either through a licensed antigen-presenting cell or in the direct presence of CD4+ T cells, CD8+ T cells must make local contact with pathogen-infected host cells. This is critical since their cytotoxic effector function must be focused on the pathogen-infected target cell, with minimal engagement of other tissue cells. Thus, CD8+ T cells must recirculate and localize to sites of infection. The *in vivo* localization or homing of CD8+ T cells is controlled by selectin-addressin interactions, which mediate initial attachment to vascular endothelium and permit rolling, and by integrins that facilitate firm adhesion. For example, CD8+ T cells recirculate from the blood into lymph nodes through their expression of L-selectin, which engages an addressin in the high endothelial venules of lymph nodes [7]. In contrast, CD8+ T cells with the potential to enter the intestine may express either the alpha4-beta7 integrin, or the chemokine receptor CCR9 [8], [9]. While CD4+ T cell help is required for full CD8+ T cell function [10], it is less clear whether CD4+ T cell help also participates in CD8+ T cell localization to sites of effector function.

In the present study, we addressed the significance of CD4+ T cell help in CD8+ T cell localization, using bioluminescent imaging of Luciferase-transgenic CD8+ T cells. The expression of a transgene encoding firefly Luciferase, and controlled by a strong T cell-specific promoter, allowed us to determine the biodistribution of CD8+ T cells in albino mice. In parallel, we labeled CD8+ T cells with the dye CFSE, and used it to reveal cells that had undergone multiple replication cycles, while co-staining for such cells for integrins and chemokine receptors allowed us to visualize the changes in the expression of such homing molecules that occur in dividing T cells. The use of a replication-defective virus-based vector allowed us to deposit the test antigen, ovalbumin, in skeletal muscle, while transgenic CD8+ T cells expressing an ovalbumin-specific antigen receptor made a local and systemic immune response. Antibody-mediated CD4+ T cell depletion allowed us to test the significance of “help”, delivered by such CD4+ T cells, in the CD8+ T cell response. We conclude that in addition to its other effects, CD4+ T cell help affects the biodistribution of CD8+ T cells responding to antigen.

## Results

### Biodistribution of CD8+ T cells detected using bioluminescence

To purpose of our study was to test the effect of CD4+ T cell help on the location of CD8+ T cells during an immune response to a replication-defective Herpes Simplex Virus (HSV) amplicon-based vector that encodes ovalbumin [11]. To identify the location of the CD8+ T cells in living mice, we used bioluminescent transgenic T-lux T cells expressing firefly Luciferase [12], crossed with a T cell receptor transgenic line, OT-1, specific for the ovalbumin-derived antigenic peptide of sequence SIINFEKL. These double-transgenic mice we term T-lux/OT-1 mice. To test the role of CD4+ T cells, normal B6 mice were compared with mice depleted of CD4+ T cells using anti-CD4 antibody. As an alternative approach, we used B6 mice lacking the MHC class II molecule, I-A^b^, and therefore totally lacking CD4+ T cells. In the case of mice treated with three daily injections of anti-CD4 antibody, the extent of depletion was verified using FACS analysis of peripheral blood cells. In line with others using the same antibody [13], we found that circulating CD4+ T cells were effectively deleted. In contrast, control mice treated with vehicle (saline) alone retained their CD4+ T cells.

Either anti-CD4-treated, or saline-treated control mice were given 1×10^6^ units of HSV-based amplicon vector [14] given by intramuscular (IM) injection into the right quadriceps femoris muscle. The two vectors used were an experimental vector expressing ovalbumin (HSVova), and a control vectors expressing beta-galactosidase (HSVlac). Twenty-four hours later, the mice received an intravenous (IV) injection of 1×10^6^ purified CD8+ T cells from T-lux/OT-1 T mice. At various intervals after the adoptive transfer, mice were imaged using a Xenogen-Caliper IVIS-100 instrument [11].

**Figure 1:**
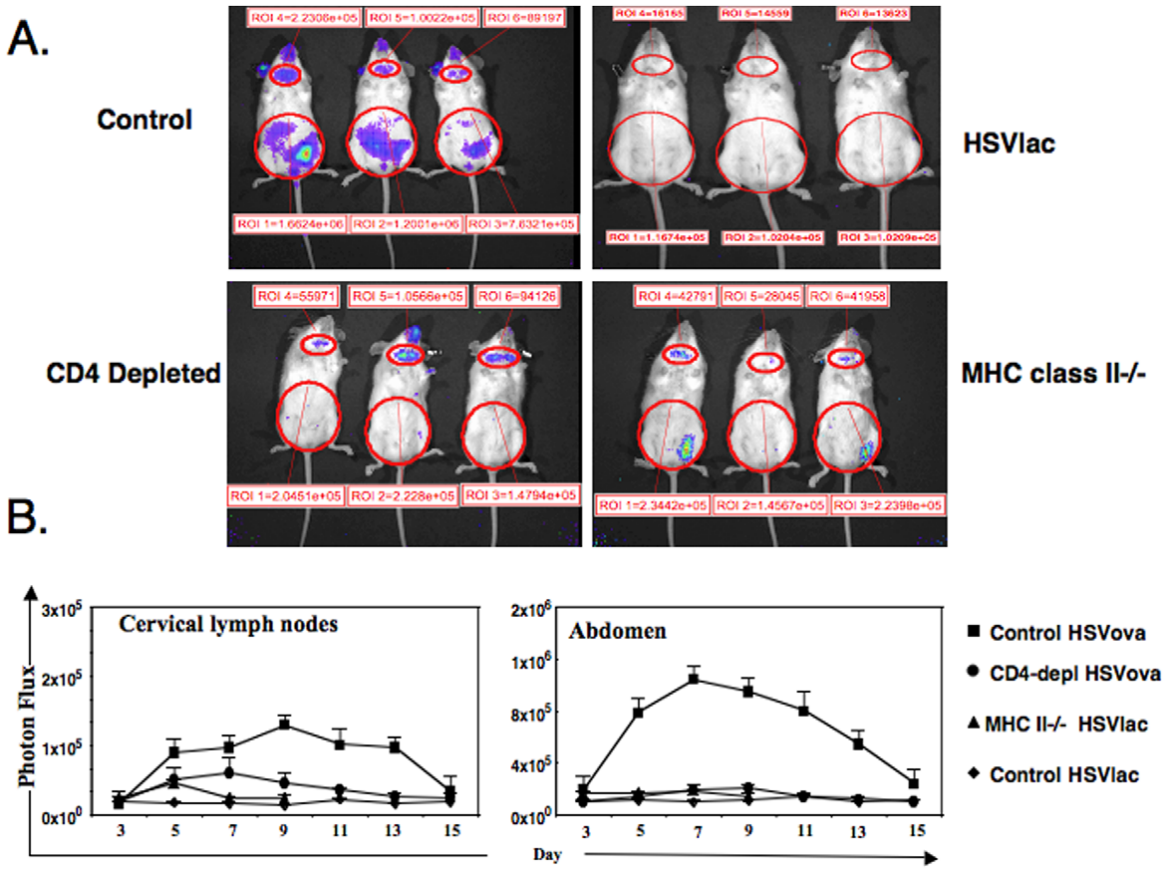
The biodistribution of T-lux/OT-1 CD8^+^ cells during a primary immune response. (A) Mice were immunized with HSVova or HSVlac, and 24 hr later infused intravenously with 1×10^4^ purified double transgenic naïve CD8^+^ T cells, and then imaged using an IVIS imager. The bioluminescent images represent the biodistribution of the CD8^+^ cells on day seven. The control group of mice had their endogenous CD4+ T cells present at the time of HSova immunization (left panel). The CD4 depleted group received three injection of anti-CD4 monoclonal antibody GK1.5 before they were immunized with HSVova (center panel). The MHC class II deficient mice lacking functional CD4+ T cells were similarly immunized (right panel). Living image software allows the superimposition of elliptical regions of interest, within which the photon flux was measured. (B) Time course of abdominal bioluminescence after administration of HSVova or HSVlac and 1×10^4^ T-lux/OT-1 CD8^+^ cells. Data are representative of 6 to 12 individually analyzed mice, and are mean ± SEM.

In normal mice, bioluminescent signal was evident in various superficial lymph nodes, including the cervical nodes, upon which we focused since these nodes were readily identifiable, bilateral, and did not drain the vector injection site. In addition, there was strong bioluminescence that developed in the abdominal region (Figure 1A, upper left panel). Our previous studies show that this is due mostly to the accumulation of cells in the mesenteric lymph nodes, with smaller signals from the milky spots in the omentum and from the spleen [11]. The bioluminescence was stronger in mice that received HSVova, compared to control mice that were given HSVlac in which the bioluminescence was below the threshold of detection (Figure 1A, upper right panel).

Deficiency of CD4+ T cells affected not only the level of bioluminescence, but in particular its biodistribution, changing the signal to a lesser extent in the cervical lymph nodes, but more strikingly in the abdomen. Thus, anti-CD4+ antibody treatment attenuated the signal (Figure 1A, lower left panel), and this effect was evident from days 5 to 13 of the response (Figure 1B). Mice that were MHC class II-deficient, and thus congenitally CD4+ T cell deficient, made still weaker but detectable responses to HSVova in the cervical lymph node, particularly on day 5 (Figure 1A, lower right panel).

While the effect of CD4+ T cell deficiency on bioluminescent signal in the cervical lymph nodes was significant but partial (Figure 1B, left panel), the effect on the bioluminescent signal in the abdomen was total (Figure 1B, right panel). In normal control mice, we detected a strong signal from the abdomen that was significantly different from the signal in control vector (HSVlac) treated mice, and maximal on day 7. In contrast, the CD4-depleted mice, there was no increase in the abdominal bioluminescence in HSVova treated mice. Similarly, there was no detectable bioluminescent signal above background in the abdomen of MHC class II deficient mice given the HSVova vector and bioluminescent OT-1 T cells (Figure 1A and B). Taken together, the different impact of CD4 deficiency on the antigen-dependent bioluminescence of cervical lymph nodes *versus* the abdominal region suggested that CD4+ T cells might influence the in vivo biodistribution of CD8+ T cells.

### The possibility of differential CD8+ T cell expansion

An alternative explanation for the differential effects of CD4+ T cells in cervical *versus* abdominal lymphoid structures was that the CD4+ T cells differentially affected proliferation in the two sites. To test for this possibility, 1×10^6^ purified CFSE labeled OT-1 CD8^+^ T cells were adoptively transferred into either intact or CD4+ T cell-depleted mice that had previously been given HSVova. At 72 hours after T cell administration, lymphoid tissues were harvested: the inguinal lymph nodes (ILN), mesenteric lymph nodes (MLN), cervical lymph nodes (CLN) and the spleen (SPL).

**Figure 2:**
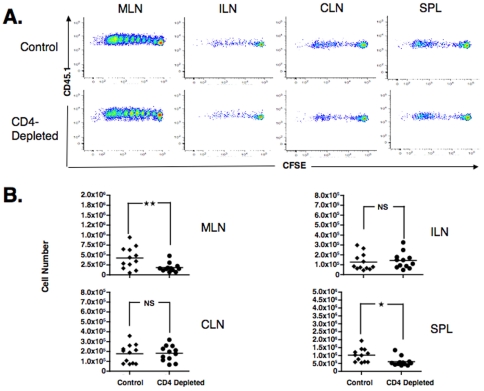
*In vivo* OT-1 CD8^+^T cells proliferation to HSVova in various secondary lymphoid organs. (A) Mice that received CFSE-labeled naïve OT-1 CD8^+^ T cell were immunized i.m. with HSVova. Three days post adoptive transfer of ova specific CD8^+^ cells, the proliferation of CFSF-labeled cells in mesenteric (MLN), inguinal (ILN) and cervical lymph nodes (CLN) and from the spleen (SPL) was visualized using flow cytometry. All dot plots were gated on CD8^+^ T cells. (B) Cell numbers obtained from MLN, ILN, CLN and SPL at day three post adoptive transfer of ova specific OT-1 T cells. There were significantly fewer OT-1 cells in CD4-depleted mice in the MLN (** p<0.013) and the SPL (*p<0.005) but not the other lymph nodes. Data are representative of six independent experiments (n = 2 mice per group for each experiment).

Activated OT-1 cells were obtained in all lymphoid tissue examined, and the pattern of proliferation of CD8^+^ antigen specific T cells from both positive control and CD4 depleted mice were similar (Figure 2A). However, the cell numbers obtained from MLN and SPL were significantly less (p<0.05) in the CD4 depleted mice, compared to control mice (Figure 2B). CD8^+^ OT-1 T cells numbers in the other lymphoid organs did not differ between the two groups (Figure 2B). This result suggests that the lack of help from CD4^+^ T cells at the time of priming did not compromise CD8^+^ proliferation, since the pattern of CFSE dilution was the same; this was also true of the down regulation of CD62L (data not shown). However, there were fewer OT-1 cells in the MLN and the spleen, both of which are sources of abdominal bioluminescence [11]. To account for the differences in CD8+ T cell abundance in the absence of CD4+ T cell help without clear differences in cell proliferation, we first considered the possibility that the CD4+ T cell help was protecting the CD8+ T cells from death. However, testing for cellular markers of apoptosis did not reveal any differences. Thus, we can best explain the effects of CD4+ T cell help on the abundance of CD8+ T cells in specific tissue sites as a result of a selective effect on CD8+ T cell homing.

### CD4^+^ T cells modify expression of gut homing molecules on CD8^+^ T cells

**Figure 3:**
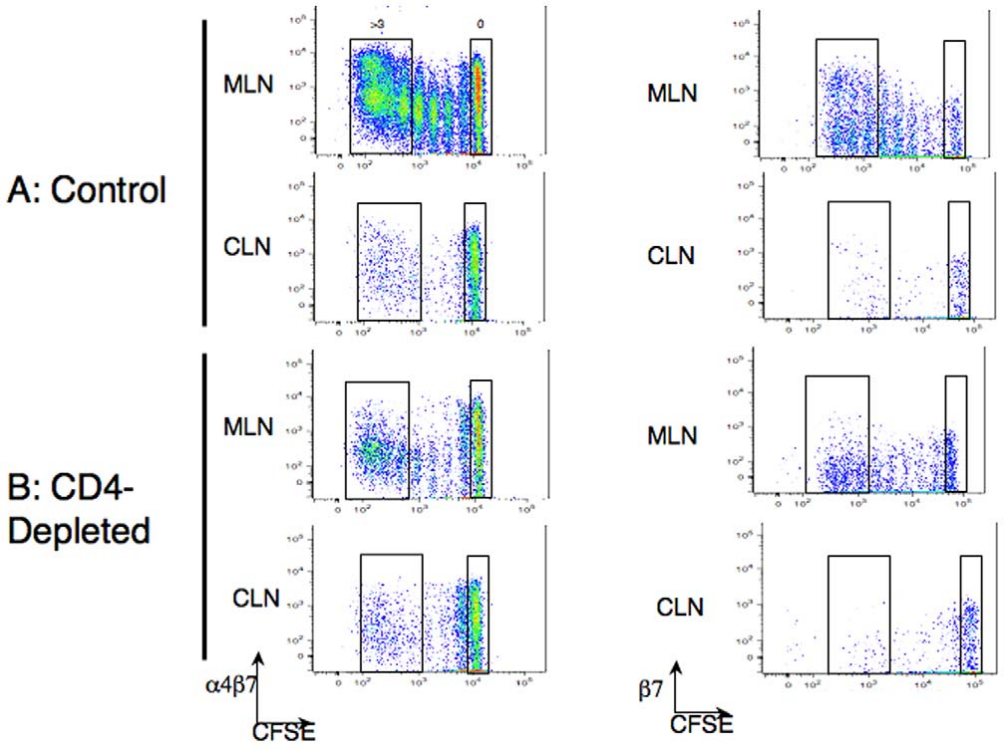
Expression of adhesion molecules on proliferating ova-specific CD8^+^ T cells after HSVova. (A) Expression of alpha4-beta7 integrin (left) or the beta7 integrin chain (right) was assessed by flow cytometry on undivided (high CFSE) ova specific CD8^+^ T cells, and on cells that had undergone more than four divisions (low CFSE), taken from MLN and CLN from control mice. Integrin expression was revealed by staining with a PE-conjugated antibody on day 4-post immunization. (B) The expression of adhesion molecules was analyzed by flow cytometry on undivided and divided CD8^+^ OT-1 cells in CD4 depleted mice. Comparing (A) with (B), there was a clear difference in the modal expression of alpha4-beta7 integrin in the MLN.

**Figure 4:**
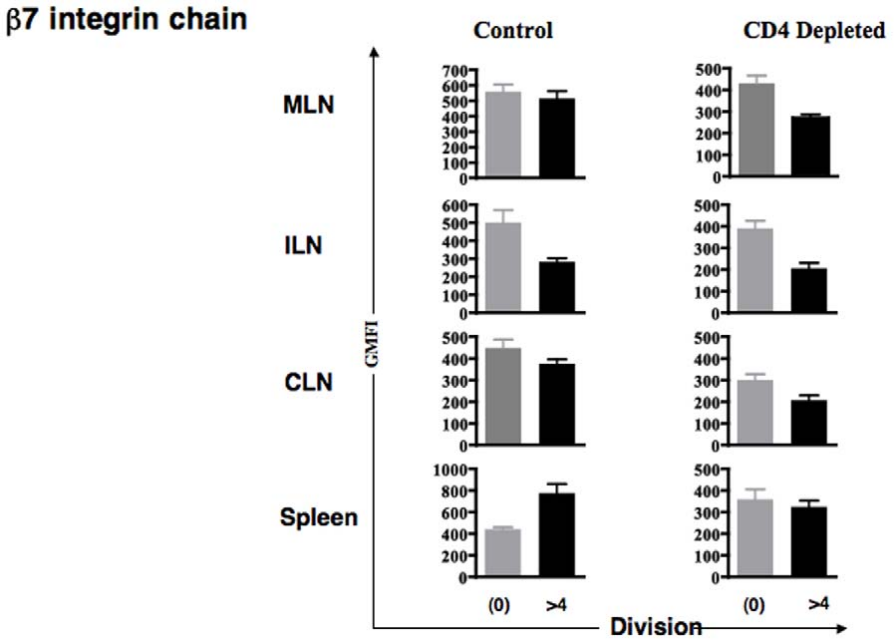
Changes in beta7 -integrin expression with cell division. Mice were immunized with HSVova i.m. and 24 hr later infused i.v. with CFSE labeled CD8^+^ T cells. Different lymphoid organs were taken out four days post HSVova. The level of beta7 -integrin expression was calculated as the geometrical mean fluorescence intensity (GMFI) and is presented as a function of undivided (0) and more than 4 divisions, based on CFSE dilution. Upregulation of beta7 integrin on CD8+ T cells from MLN was significant (p = 0.0022) in control mice but not in CD4+ depleted mice. Mice were from three experiments (n = 2 mice per group per experiment).

**Figure 5:**
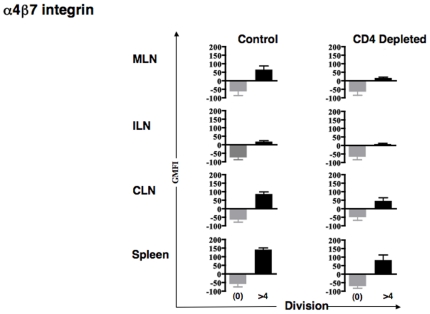
Changes in alpha4-beta7 integrin expression on dividing CD8^+^ T cells. The level of expression of alpha4-beta7 integrin was calculated as the geometrical mean fluorescence intensity and is presented as a function of cell division for each individual mouse. In the MLN, there were increase in alpha4-beta7 integrin expression in both control and CD4+ treated mice, but the increase in GMFI was significantly greater in control mice (p = 0.0325). Data are from three experiments (n = 2 mice per group per experiment). A GMFI of >0 implies fluorescence intensity lower than the unstained control.

Antigen engagement by naïve T cells results in changes in the expression of homing and adhesion molecules. In particular, activated T cells interacting with intestine-derived dendritic cells selectively increased their expression of alpha4-beta7 integrin [15]. Since our experimental design made use of the adoptive transfer of naïve OT-1 T cells, we were able to distinguish cells that have undergone multiple division cycles using the dye CFSE. Therefore, to test whether CD4^+^ T cell help could be influencing the biodistribution of CD8^+^ T cell *via* effects on these molecules, we analyzed the expression of gut homing molecules on CFSE-labeled OT-1 T cells in ILN, CLN, MLN and SPL within 3 days after adoptive transfer (Figure 3, left panels). There was increased expression of the alpha4-beta7 integrin in the OT-1 cells that had divided several times in MLN and spleen from normal control mice, but less increase in CD4 depleted mice (Figure 3, left panels). We compared geometric mean fluorescence intensity (GMFI) from several round of divisions in control mice *versus* CD4-depleted mice using a Mann Whitney test. The beta7 integrin chain up-regulation was statistically significant for OT-1 cells in the MLN in normal control mice (P = 0.0022; Figure 4). The alpha4-beta7 integrin heterodimer, specifically associated with gut homing, was also upregulated on the CD8^+^ T cells that divided more than 4 times in MLN (Figure 5). In this case the expression was increased overall in both control and CD4-depleted mice. We therefore compared the division-associated differences in GMFI between control and CD4+ depleted mice. We concluded that the upregulation of this integrin was significantly greater in HSVova treated control mice than in HSVova treated CD4-depleted mice (p = 0.0325). In view of this result we also tested the expression of CCR9, which is frequently up-regulated on gut homing lymphocytes. The CCR9 molecule was indeed strongly up-regulated in OT-1 T cells after cell division (Supplementary Figure S1). However, unlike the alpha4-beta7 integrin, there was no clear effect of CD4+ T cell depletion on CCR9 upregulation.

These data show that activated antigen specific CD8^+^ T cells acquired tissue specific homing molecules within the lymph nodes 4 days after ova inoculation. In control mice, the ability to re-localize into intra-abdominal lymphoid organs increased after several rounds of division, in parallel with the acquisition of tissue specific homing molecules such as the beta7 integrin chain, and the alpha4-beta7 integrin heterodimer. The ability of such CD8^+^ T cells to up-regulate these gut homing markers was impaired in CD4-deficient mice.

### The effect of CD4+ T cell help in the CD8^+^ T cell memory response

To test whether CD4+ T cells, present during priming, had effects on CD8+ T cell location that persisted in the memory phase, we re-challenged the experimental mice with SIINFEKL peptide 45 days after priming. Mice were imaged before, and then at time intervals after the secondary challenge with peptide, which was given IP on three constitutive days, each dose being 25 nmol of SIINFEKL diluted in 100 µl of saline. In the cervical lymph nodes, we did not observe clear effects of CD4+ T cell depletion on CD8+ T cell localization. Thus, the bioluminescent signal appeared at first to be attenuated (but this effect was not statistically significant, p = 0.248) in mice that received anti-CD4 antibody treatment on day 2 post SIINFEKL (day 49 post priming) but 2 days later, on day 52, it reached a level comparable to control mice given the HSVova vector, and was in excess of control mice that had received the HSVlac control vector (data not shown). Overall, the bioluminescent signal in the CLN was not significantly different between the anti-CD4 treated and the untreated control mice (p = 0.4).

**Figure 6:**
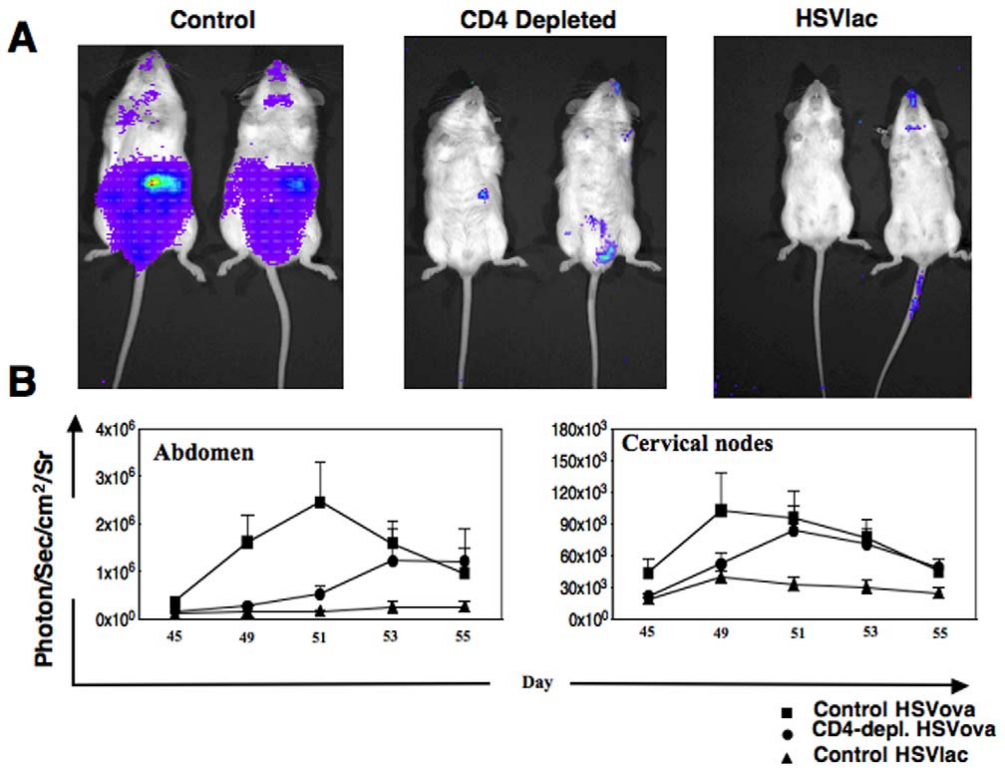
Bioluminescent imaging of the memory response. (A) Mice were re-challenged with 25 nM SIINFEKL peptide 45 days post priming and biodistribution of T-lux/OT.1 cells was determined using *in vivo* imaging. The images represent individual mice from day four-post challenge (51 days post priming). Bioluminescent signals in HSVova mice were distributed mostly in the abdominal region, in contrast to the CD4 depleted mouse. (B) Time course of abdominal bioluminescence of T-lux/OT.1 cells after administration of SIINFEKL peptide. The data are from two experiments (n = 3 mice per group in each experiment). In HSVova-treated positive control mice, the photon flux peaked by day 6 post secondary challenge; however the signal from CD4 depleted mice was significantly lower at early times but peaked 8 days later.

In contrast to the lymph nodes, the effect of CD4+ T cell depletion during priming was striking during the recall responses in the abdominal lymphoid organs. In the HSVova positive control mice, the photon flux in the abdominal region peaked four day after challenge (day 51 post priming; Figure 6A and 6B), and then declined towards the control level (HSVlac) by day eight. The photon flux in the abdominal region peaked approximately 2-fold higher than the primary response (compare Figure 1B with Figure 6B), and the appearance of CD8+ T cells in abdominal sites occurred earlier. In contrast, the CD4 depleted mice had much lower bioluminescent signals measured in the abdominal region on day four; this difference was statistically significant (p = 0.04). However, the most striking feature of the secondary response was the delay in the response in the abdominal region in CD4-depleted, compared to control HSVova-treated mice. The kinetics of this response was in fact very similar to the accumulation of bioluminescence in the abdomen in the normal control mice during the primary response (compare Figure 6 with Figure 1). Thus, CD4+ T cells were necessary for the localization of CD8+ T cells to the abdomen both during the primary response, and during a secondary response.

## Discussion

The data presented here show that CD4+ T cells influence the biodistribution of CD8+ T cells. This was shown using HSV-based amplicon vectors, given IM into the quadriceps femoris, as the antigen delivery vehicle. The CD8+ T cells were double-transgenic, expressing both an antigen-specific T cell receptor that recognizes an ovalbumin-derived peptide, and firefly Luciferase [11]. An injection of the substrate, luciferin, resulted in emission of visible light, which revealed the location of the CD8+ T cells. The technique was performed under light general anesthesia, permitting the individual mice to recover, and thereby minimizing the number of mice required for a time-course study. When CD4+ T cells were depleted using antibody, or were absent in gene-targeted mice, there was reduced overall CD8+ T cell abundance, but superimposed on this was a distinct effect on CD8+ T cell location. Specifically, there was a modest loss of bioluminescent signal from the cervical lymph nodes, but a total loss of signal in the abdominal region. In a previous study, we showed that abdominal bioluminescent in this experimental model is due mainly to the mesenteric lymph nodes [11]. Therefore, in the present experiments, the presence or absence of CD4+ T cell help was modulating the localization of CD8+ T cells into the mesenteric nodes.

The localization of activated CD8+ T cells to the mesenteric nodes after several days in the immune response to a replication-defective vector challenge in the hind limb is unlikely to be due to migration of either antigen or antigen-presenting cells to these nodes. A recent study shows that a tracer dye given into the hind foot localizes first to the popliteal and the inguinal lymph nodes, and subsequently drains to the iliac nodes [16]. These iliac lymph nodes are indeed deep abdominal structures, but they do not constitute the majority of the bioluminescence in an anti-HSV amplicon response, as we showed previously [11]. Therefore, in the presence of CD4+ T cells the localization of the CD8+ T cells to the abdomen in most likely driven not by antigen, but by cell-intrinsic homing properties.

This change in CD8+ T cell abundance in different tissue sites could have resulted from several mechanisms. We tested for, and excluded a potential mechanism based on differential T cell division in the cervical *versus* mesenteric nodes, using the dye CFSE to stain T cells and dilution of the dye to indicate cell proliferation. We also tested the possibility that CD4+ T cell help was influencing the expression of cell surface adhesion molecules, and found that in the presence of CD4+ T cells, the dividing CD8+ T cells increased the expression of the alpha4-beta7 integrin, which is associated with homing of CD8+ T cells to the intestine. This was confirmed using a second antibody specific for the beta7 integrin chain alone. Other markers of cell activation or homing potential were not so clearly affected. We conclude that the CD4+ T cells were most likely modulating CD8+ T cell localization through an effect on homing, mediated in particular by alpha4-beta7 integrin.

The immune responses of CD8^+^ T cells are sometimes strictly CD4^+^ T cell help-dependent, and sometimes more-or-less CD4^+^ help-independent, based on the nature of the immune stimulus. Help-independent responses are typical of those to an infectious agent, such as the bacterium, *Listeria monocytogenes* [17], while the help-dependent response was typical for non-inflammatory antigens, such as minor histocompatibility antigens [18], [19]. A consensus is emerging in which, in the absence of the innate immune activation signals delivered to antigen presenting cells, help from CD4^+^ T cells is a requirement for an effective CD8^+^ T cells response. The level of inflammation involved in different experimental model systems may also modulate the help-dependence of CD8^+^ T cells.

The interaction of CD4+ T cells with CD8+ T cells may be mediated via dendritic cells, which are central in naïve T cell activation under most circumstances. This model that explains this 3-cell interaction, termed “licensing”, was proposed more than 10 years ago[20], [21], [22]. In this model, interaction of CD40 with its counterpart, CD40L, which is expressed on CD4^+^T cells, leads to maturation of dendritic cells. However, in the case of viruses, the pathogen can induce signals *via* Toll-like receptors without help from CD4 for maturation of dendritic cells [23], [24]. Such dendritic cells have the ability to distinguish the pathogen signals and influence the characteristics of the T cell responses that they induce [25]. Several studies using soluble antigen, such as ovalbumin, have reported tissue-specific combinations of adhesion molecules and chemokine cues resulting in selective recruitment of CD4^+^ T cells to either the gut or the skin [26], [27]. These primed CD4^+^ T cells of the intestinal lymph nodes have up-regulated expression of integrin alpha4-beta7; however, CD4^+^ T cells that were primed in the skin lymph nodes have a high expression of P-selectin ligands [28]. In the case of CD8^+^ T cells, *in vitro* priming studies reported that dendritic populations are important in determining whether gut or skin homing phenotypes are on the primed CD8^+^ T cells [15], [29], [30]. Thus, bone marrow-derived peptide-pulsed dendritic cells injected *via* different routes of delivery resulted in up-regulation of different homing phenotypes on primed CD8^+^ T cells *in vivo* [29]. In the context of the present study, we therefore think it is likely that dendritic cells in the popliteal and/or inguinal draining lymph nodes impose upon OT-1 CD8+ T cells the changes in alpha4-beta7 integrin expression that predispose the cells to homing to abdominal lymphoid structures.

The role of CD4^+^ T cell help in generating memory CD8^+^ T cells also varies between different experimental models. Early reports suggested that CD4^+^ T help is essential for the generation of functional CD8^+^ T cell memory to most antigens [13], [31], and that un-helped CD8^+^ T cells make a poor memory response upon secondary challenge [31]. However, in other experiments, un-helped CD8^+^ T cells had the ability to proliferate after secondary challenge [32]. As in the case of primary responses, these discrepancies best fit a model in which accessory signals determine the requirement for CD4^+^ T cell help.

The concept that CD4+ T cell help for CD8+ T cells is mediated by licensing of dendritic cells implies the recognition of antigen that is presented to the CD4+ T cell by the dendritic cell. Thus, the antigen specificity of the CD4+ T cell would be crucial for this kind of help. Our CD4+ T cell depletion experiments did not distinguish between antigen-specific and non-specific help, and it may have been better not to exclude either. The role of antigen-specificity in CD4+ T cell help for CD8+ T cells is ambiguous; one study showed that the presence but not the specificity of CD4+ T cells was important in long-term CD8+ T cell memory [33]. Another showed that heterospecific CD4+ T-helper cells could deliver help to CD8+ T cells [34].

The observation that a brief antigen exposure could result in clonal expansion of naive CD8^+^ T cells, followed by effector functions and memory cells, gave rise to the concept of “programming” [35]. In this concept, a fully-activated naïve CD8^+^ T cell needs no further antigenic signal to execute its full differentiation program. It was suggested that the CD4^+^ T help was crucial at an early time point, during initial CD8^+^ T cell priming, and that this could influence the later development of memory CD8^+^ T cells [13]. However, not all experiments supported a programming model for CD4^+^ T cell help; other studies were more consistent with the concept that CD4^+^ T help was necessary for maintaining, rather than programming CD8^+^ T cell memory [33].

After priming, activated T cells enter the circulation and migrate to many tissues, including secondary lymphoid organs and non-lymphoid tissue. Studies by Masopust and colleagues [36] show that virus specific effector and memory T cells are present in a wide range of host tissues, regardless of the route of viral infection. In our data, we observed an effect of CD4+ T cell depletion during the initial priming of CD8+ T cells that was manifest in the memory phase, almost 60 days later. This was a time at which the CD4+ T cells had recovered from antibody depletion. Therefore, we think these effects fit with the idea of programming. Our working hypothesis is that CD4+ T cell help programs CD8+ T cells for specific homing, particularly to the mesenteric lymph nodes. In the absence of such programming, primed CD8+ T cells do not go there.

On the basis of the data presented here, we speculate that CD4+ T cell help for CD8+ T cells entails an element that we propose to call “programming for location”. While we have observed this only in a vector-driven model, it may be a general feature of helper-dependent CD8+ T cell responses. If this model is correct, programming for location may contribute to why CD4+ T cell help is important in effective CD8+ T cell-mediated immunity, for example to pathogens such as Hepatitis C Virus, in which some individuals eradicate infection, others fail to do so, and the difference is closely associated with the presence or absence of a CD4+ T cell response.

## Materials and Methods

### Ethics Statement

All animal experiments were approved by the Institutional Animal Care and Use Committee.

### Mice

C57BL/6J-Tyrc-2J/J albino mice were purchased from Jackson Laboratory (Bar Harbor, ME, USA) and were kept in the University of Rochester SPF vivarium. MHC class II-deficient mice were obtained from Taconic Farms (Germantown, NY, USA) and were backcrossed three times to C57BL/6L-tyrc-2J/J in order to have MHC class II deficient mice with an albino coat. For *in vivo* imaging experiments, the lack of melanin pigment in these albino mice increased the sensitivity of detection substantially. Luciferase-expressing transgenic mice (T-lux mice) [12] were a kind gift from Dr. Casey Weaver (University of Alabama Birmingham, AL, USA). A colony of OT-1 TCR transgenic mice [37] was maintained on a CD45.1 background. Crossing H-2^b^ T-lux mice with OT-1 TCR transgenic mice generated double-transgenic mice, in which the CD8^+^ T cells were specific for the SIINFEKL peptide of ovalbumin and all T cells expressed the Luciferase reporter gene. These mice were CD45.1 x CD45.2 heterozygous, and are termed T-lux/OT-1. T cells from T-lux/OT-1 were identified in host mice, based on CD45.1 expression.

### Flow cytometry

Mice were typed using the allotypic marker CD45.1 (clone A20) PE conjugated, TCR Valpha2 (clone B20.1) PE-conjugated, Vbeta5 (clone MR9-4) FITC conjugated and CD8α (clone 53-6.7) PerCP conjugated antibodies from BD PharMingen (San Jose, CA, USA). Data were acquired using a FACSCalibur® (Becton Dickinson Immunocytometry System, San Jose, CA, USA).

Antibodies to the murine alpha4-beta7 heterodimer (clone DATK32), and beta7 (clone M293) integrin chains were from BD Pharmingen (San Jose, CA, USA). Both integrin antibodies were PE-conjugated. Lymphoid organs were harvested on day three-post adoptive CD8^+^ T cell transfer; these were the inguinal lymph nodes (ILN), mesenteric plus lumbar nodes (MLN), cervical lymph nodes (CLN) and the spleen (SPL). These tissues were homogenized mechanically and cells re-suspended in Hanks Balanced Salt Solution (HBSS) containing 5% heat inactivated fetal bovine serum. Cell suspensions were counted, and incubated with Live/DEAD stain (Invitrogen, Eugene, OR, USA) before surface marker antibody staining. Subsequently, cells were washed with PBS and re-suspended in PBS for FACS analysis using a LSRII™(BD Biosciences, San Jose, CA, USA). Doublets were excluded based on a gate on forward height *versus* area, and viable lymphocytes were identified using a gate based on forward and side scatter, and by using the Live/Dead staining reagent.

### Bioluminescence imaging and image analysis

Mice were anesthetized with intraperitoneally (i.p.) injection of ketamine (100 mg/kg body weight) and xylazine (10 mg/kg body weight) and then given an i.p. injection of D-luciferin (214 µg/g body weight; Xenogen-Caliper corp., Alameda, CA, USA). After 5 min, the mice were positioned in the imaging chamber (IVIS-100 series) for data collection. Briefly, the IVIS-100 series consists of a cooled CCD camera mounted on a light-tight chamber, a cryogenic unit for camera refrigeration and a computer for image collection and analysis. The acquisition time was 5 min both for the primary response and for the secondary challenge. Bioluminescence data were obtained using medium binning, for high resolution but at the cost of loss of some sensitivity. Relative intensities of emitted light were represented as pseudocolor images ranging from red (most intense) to blue (least intense). Gray scale photographs and the corresponding pseudocolor images were superimposed with Living Image (Xenogen-Caliper) and Igor (waveMetrics, Lake Oswego, OR, USA) image analysis software. Signal emitted by regions of interest (ROI) was measured and data were expressed as photo flux and quantified as photon sec^−1^ cm^−2^ sr^−1^. The steradian (sr) term refers to photons emitted from a unit solid angle of a sphere. Data were shown as mean ± SEM. The machine background was subtracted electronically both from the images and from the measurements of photon flux.

### Herpes Simplex-based Amplicon Vectors

Helper-free herpes simplex virus type 1 (HSV-1) based amplicon vectors encoding the test antigen ovalbumin were assembled in the Center for Neural Development and Disease, Amplicon Vector Production Core at University of Rochester (Rochester, NY, USA). The HSV amplicon consists of the HSV-derived immediate-early 4/5 promoters, which drive the expression of the ova gene (termed HSVova) or control HSV amplicon expressing beta-galactosidase (termed HSVlac). The amplicon can transduce a variety of tissues including post-mitotic neurons [38]. Amplicon stock construction and the estimation of vector concentration was described previously [14], [39], [40].

### Immune Response to HSV Amplicons

We previously established an in vivo imaging protocol in which the immune response of adoptively transferred, double transgenic CD8^+^ T cells (T-lux/OT-1) against ova was visualizes non-destructively after IM inoculation of HSV-based amplicons [11]. Activated double transgenic CD8^+^ T cells, T-lux/OT-1, responded to HSVova and could be detected in injection site, inguinal lymph node and intra-abdominal organs. We could detect dispersion of the cells systemically, but a majority of cells remained within abdomen.

To document the duration of antigen expression, purified transgenic CD8^+^ T cells were transferred IV either 1 or 13 days after HSVova injection. Measurement of bioluminescent signal from intra-abdominal structures at 1 day post immunization resulted in maximal photon flux in the abdominal region by day 5 post cell transfer. In contrast there was no signal above baseline in immunized mice that received T-lux/OT-1 CD8^+^ T cells 13 days after HSVova (data not shown). These data show that expression of the HSV-encoded Ag was transient, even without exogenous T cells. The disappearance of antigen within 13 days is consistent with a previous report using HSV amplicon expressing Luciferase, in which the maximum signal from IM inoculation of HSVlac was at 24 hr and it declined rapidly thereafter [41].

### Adoptive Transfer and cell proliferation assay

Peripheral lymph node and spleen cells were harvested from either OT-1 or T-lux/OT-1 mice. A single cell suspension was prepared by mechanical homogenization of tissues, and red blood cells were removed using Lympholyte M (Cedarlane Laboratories, Burlington, NC, USA). Naive purified CD8^+^ T cells were obtained using negative selection *via* Miltenyi MACS purification kits (Miltenyi Biotech Inc., Auburn, CA, USA) according to the manufacturers' protocol. To detect T cell proliferation in vivo, purified CD8^+^ T cells were labeled with 5 micromolar CFSE (Molecular Probes, Eugene, OR, USA) at 37°C for 10 min and washed three times before IV transfer to the recipients. In the bioluminescent imaging experiments, recipient mice were given 1×10^4^ purified double transgenic CD8^+^ T cells, and for *in vivo* proliferation 1×10^6^ purified transgenic CD8^+^ T cells.

### Depletion of CD4^+^ T cells

GK1.5 (anti-CD4) hybridoma was grown in BD serum-free medium, then transferred to BD CELLine culture system (BD biosciences, Sparks, MD). Culture supernatant was harvested and pooled for antibody production. Pooled supernatant was purified by 50% ammonium sulfate precipitation, and dialysis. Antibody titer was measured by ELISA, and the antibody was sterilized by filtration. CD4^+^ cell depletion was achieved by i.p. injection of 50 micrograms of purified GK1.5 in 0.1 ml of PBS, given on three constitutive days prior to immunization. The effectiveness of depletion was monitored by staining peripheral blood with a non-competing anti-CD4 mAb (clone RM4-4) and anti CD3 (Clone 145-2C11) using a FACSCalibur® flow cytometer and CellQuest software. Both antibodies were purchased from BD Pharmingen (San Jose, CA, USA).

### Peptide

The SIINFEKL peptide, corresponding to residues OVA _257–264_, was purchased from New England Peptide (Fitchburg, MA, USA) and mice were re-challenged with daily i.p. injections of 25 nmol of peptide for 3 days.

### Statistical Analysis

Bioluminescence data were pooled from multiple experiments and expressed graphically as mean ± SEM. The Mann Whitney test was used to measure the significance of difference observed between the experimental groups. Statistical analysis was performed using Prism software (GraphPad Software, Inc., La Jolla, CA, USA).

## Supporting Information

Figure S1 The absence of CD4+ T cell help does not affect the up-regulation of the CCR9 chemokine receptor. CFSE versus CCR9 staining was used to identify OT-1 T cells that had divided 4 or more times. The geometric mean fluorescence index (GMFI) was increased in dividing T cells in all of the lymph nodes examined, but unlike the case for alpha4-beta7 integrin, there was no significance difference between intact positive control mice and mice that were CD4-depleted.
(TIF)
