# Supplementary figures and images for: CD4+ T Cell Effects on CD8+ T Cell Location Defined Using Bioluminescence
Source: PLoS One. 2011 Jan 20;6(1):e16222. doi: 10.1371/journal.pone.0016222 (PMC3024405; doi:10.1371/journal.pone.0016222)

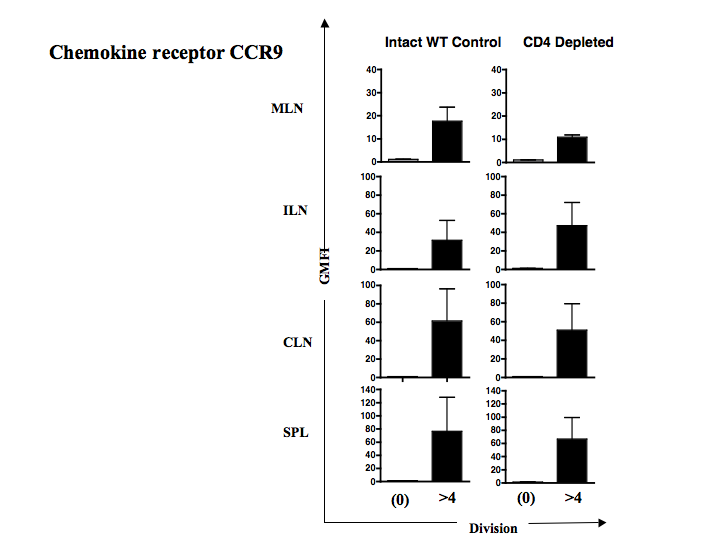

Supplement: Figure S1 — The absence of CD4+ T cell help does not affect the up-regulation of the CCR9 chemokine receptor. CFSE versus CCR9 staining was used to identify OT-1 T cells that had divided 4 or more times. The geometric mean fluorescence index (GMFI) was increased in dividing T cells in all of the lymph nodes examined, but unlike the case for alpha4-beta7 integrin, there was no significance difference between intact positive control mice and mice that were CD4-depleted. (TIF) [file pone.0016222.s001.tif]
